# Roles and reimbursement of pharmacists as South Africa transitions towards Universal Health Coverage (UHC): An online survey-based study

**DOI:** 10.1371/journal.pone.0257348

**Published:** 2021-09-23

**Authors:** Vivian Naidoo, Fatima Suleman, Varsha Bangalee

**Affiliations:** Discipline of Pharmaceutical Sciences, Westville Campus, University of KwaZulu-Natal, Durban, South Africa; Yale University, UNITED STATES

## Abstract

**Background:**

The implementation of Universal Health Coverage in SA has sought to focus on promoting affordable health care services that are accessible to all citizens. In this regard, pharmacists are expected to play a pivotal function in the revitalization of primary health care (PHC) during this transition by the expansion of their practice roles.

**Objectives:**

To assess the readiness and perceptions of pharmacists to expand their roles in an integrated health care system. To determine the availability and pricing of primary health care services currently provided within a community pharmacy environment and to evaluate suitable reimbursement for the provision of such services by a community pharmacist.

**Methods:**

Community pharmacists’ across SA were invited to participate in an online survey-based study. The survey consisted of both open- and closed-ended questions. Descriptive statistics for closed-ended questions were generated and analysed using Microsoft Excel^®^ and Survey Monkey^®^. Responses for the open-ended questions were transcribed, analysed, and reported as emerging themes.

**Results:**

Six hundred and sixty-four pharmacists’ responded to the online survey. Seventy-five percent of pharmacists’ reported that with appropriate training, a transition into a more patient-centered role might be beneficial in the re-engineering of the PHC system. However, in order to adopt these new roles, appropriate reimbursement structures are required. The current fee levied by pharmacists in community pharmacies that offered these PHC services was found to be lower to that recommended by the South African Pharmacy Council; this disparity is primarily due to a lack of information and policy standardisation. Therefore, in order to ensure that fees levied are fair, comprehensive service package guidelines are required.

**Conclusions:**

This study provides baseline data for policy makers on pharmacists’ readiness to transition into expanded roles. Furthermore, it can be used as a foundation to establish appropriate reimbursement frameworks for pharmacists providing PHC services.

## Introduction

The South African government has begun to develop new health policies to aid the transitioning towards Universal Health Coverage (UHC) with the publication of the Bill for National Health Insurance (NHI). The NHI is centred on the principle of the constitutional right of all South African citizens; this being the right to access quality health care services. Under the NHI, health care services will be distributed effectively, equitably, efficiently, affordably and appropriately focusing on progressive universalism, social solidarity, equity and health as a public, social and noteworthy investment [1].

South Africa’s (SA’s) current primary health care (PHC) system is a remnant of the past apartheid era. Introductory NHI activities have therefore focused on re-engineering this system and the referral processes to ensure the equitable distribution of health services [2]. Primary health care by definition addresses the main problems experienced in a community and is aimed at providing curative, preventative, promotive and rehabilitative services. Initiatives to strengthen PHC will therefore ensure that South Africans would have access to healthcare services covered by NHI in close proximity to where they reside. It has been projected that the proper execution of PHC services could eliminate 21% to 38% of the burden of premature mortality and disability in children under 1–5 years of age, and 10% to 18% of this burden in adults; thus reducing the strain on higher levels of the health care system [1].

Community pharmacists’ are generally the first port of call for patients seeking medical advice and are therefore most likely to provide services that address the primary health care needs within these communities [3]. PHC services such as blood glucose and cholesterol screening and monitoring, immunization, drug utilization reviews and selective reproductive services are just some of the services that can be offered within community pharmacies by a pharmacist [4]. Countries such as France, Belgium, Norway and those making up the United Kingdom have significantly improved the efficiency of their health care referral pathways by incorporating pharmacists’ as an integral point of care within the PHC environment [5–9].

The scope of practice and tertiary education of a pharmacist encompasses a diversity of skills and knowledge; despite this, several of these skills remained untapped in SA. Therefore, with the implementation of the NHI, the conventional role of a pharmacist is anticipated to change; pharmacists’ will be expected to take on a more integrated role by transitioning from a more product-centred focus to a patient-centred one. Increasing their involvement at a PHC level would subsequently benefit other levels of health care by reducing the workload and strain on these facilities, as patients can be managed at a primary level, stabilized and monitored on treatment. This is particularly important for patients with non-communicable diseases requiring long-term treatment [10]. Pharmacists are also comprehensively educated on the standard treatment guidelines (STG) as part of their university curriculum. The STG is a framework utilized within the South African healthcare system aimed at promoting rational and safe drug use by assisting providers with both pharmaceutical and non-pharmaceutical treatment strategies for ailments at all levels of care [11]. These guidelines are made available to South African healthcare professionals on both written and online platforms. Some tertiary institutions have also started to offer an advanced Primary Care Drug Therapy (PCDT) course that enables a pharmacist to diagnose and prescribe medication within their community according to an approved list of conditions. The course is completed over a two-year period at an accredited university and comprises of modules, workshops, case study and examination assessment methods. The benefit of this course enables pharmacist to develop their clinical skills and professional capabilities to provided more comprehensive pharmacist-initiated therapy at a PHC level. Furthermore, pharmacists can enhance their pharmacotherapy and pharmaceutical care knowledge along with their clinical skills [12]. Therefore, the repositioning and repurposing of pharmacists’ roles and utilization of previously untapped skills will significantly improve the functionality of the PHC health care system [13].

Internationally, pharmacist involvement in providing an advanced patient care package has become increasingly prevalent. In New Zealand, The United States and The United Kingdom collaborative prescribing practices have been implemented with the incorporation of a pharmacist as a prescriber [14–16]. Some countries such as The United States, Portugal, Ireland and The United Kingdom have certified pharmacists as providers for immunization programs [15]. In Canada, pharmacists have been repositioned to providing a more collaborative primary healthcare platform that focuses on direct patient care and the management of chronic conditions [15, 17]. Countries such as France, Belgium, Norway and The United Kingdom have implemented drug utilization review services such as the new medicine service or medicine therapy management that is primarily provided by a pharmacist. The implementation of such services has resulted in massive cost-savings, improved patient adherence and access to healthcare [5, 7, 9, 18]. Therefore, this presents the potential for pharmacists as primary care providers in SA to improve medication adherence in patients and reduce the burden and costs of complications to the health system. With that said, it is evident that patients will most likely benefit from these changes, however, some concerns have been identified. This includes the development of proper communication channels with medical practitioners so that a patient’s health and pharmacotherapy is managed appropriately [15]. Another concern is the willingness of medical practitioners to engage as concerns surrounding profit and liability issues have been raised [15].

Within the context of expanding the roles of pharmacists in SA, ample consideration needs to be given to the remuneration for these services, to ensure that they are feasible and fair so that pharmacists can provide these necessary services sustainably. Currently, South African community pharmacists (refers to those operating in the private sector) are remunerated by levying a dispensing fee which is based on the tiered Single Exit Price (SEP) of a medicine. However, with the NHI roll-out, remuneration of pharmacists is still unclear, and remains an area of concern for those pharmacists who are willing to be incorporated into the public-private care partnership. Furthermore, an increased level of clinical and experiential training may be required and should be incorporated within the tertiary curriculum so that pharmacists can take on a more integrated role [19]. Therefore, with appropriate training, guidelines and reimbursement policies, pharmacists can be incorporated into the primary health care service package, but their readiness to do so has not been explored.

The objectives of this study were to:-

- To assess the readiness and perceptions of pharmacists to take on the envisioned expanded roles and responsibilities in an integrated health care system such as NHI.
- To determine the availability and pricing of the PHC services currently provided within a community pharmacy environment; and to assess perceptions as to the suitability of reimbursement for the provision of such services by a community pharmacist.

These findings can provide preliminary evidence for policy makers to develop a clear framework for incorporating pharmacists as key players in PHC services. Furthermore, the findings of this study provides direction for pharmacists, aiming at expanding their professional capabilities.

## Methods

### Study design and population

The study was conducted from January-July 2020 in the form of an online survey. In this study, a community pharmacist can be defined as a healthcare provider within a community environment, were drug and health related services are freely accessible and provided based on the specialised knowledge of a pharmacist for the purposes of promotion of effective treatment management and other health related problems [20]. Therefore, based on the objectives of this study, pharmacists practicing within a community setting across South Africa (SA) formed the study population.

### Ethical clearance

Ethical clearance to conduct the study was obtained from the Biomedical Research Ethics Committee at the University of KwaZulu-Natal, Durban, South Africa (BREC Ref No: BE625/17). Participants were notified that their participation was voluntary and that they could withdraw at any stage. Every participant was briefed in full on the aims, objectives and procedures of the study and only proceeded upon completing an online informed consent declaration. Participants anonymity was maintained through-out the study. Participants were given communication channels of all researchers should they have any concerns.

### Study questionnaire

An online survey was utilized to obtain data as it has exhibited significant validity and reliability as a research tool for both qualitative and quantitative health and social science research [21, 22]. The survey was developed after reviewing current literature and the gaps that the study aimed to fulfil in assessing pharmacists readiness and reimbursement frameworks with the implementation of NHI. The survey was composed of twenty-two questions divided into three sections. Section A consisted of four statements relating to the demographics of the participants i.e. age, sex, fields of study, geographical area of practice, languages spoken. Section B consisted of six statements that were used to determine the availability and pricing of PHC services currently provided in community pharmacies, with two of them being specifically focused on evaluating current and proposed reimbursement for the provision of these services. Section C consisted of 10 statements that sought to obtain pharmacists perceptions, opinions and their level of readiness to provide these services within the NHI PHC service package. The survey comprised of a mixture of yes/no, checklist, drop-down short responses and opened ended responses The survey was piloted amongst eight community pharmacists to ensure correctness of wording, ambiguity and logic, upon which minor amendments were made accordingly. The final questions was face validated by two academic pharmacists at the University of KwaZulu-Natal, South Africa.

### Data collection

An email listing was obtained from the South African Pharmacy Council (SAPC) for all registered community pharmacists in SA and a survey was distributed using an online platform i.e. SurveyMonkey^®^. A convenience sampling technique was employed to recruit participants. Consenting participants who did not respond were sent a reminder after 14 days to complete the online survey.

### Data analysis

The Raosoft^®^ sample size calculator was used to determine sample size for the study. Based on a total of 3440 community pharmacists, a minimum of 346 responses would be needed to achieve a 95% confidence interval. Furthermore, due to the slow uptake of the online survey a 50% response rate was expected with a 5% margin of error. The data from the online surveys were collated and the resulting data was analysed using SurveyMonkey^®^ analysis and Microsoft Excel. Quantitative data was analysed and reported utilizing a descriptive analysis approach whereas qualitative data was reported and evaluated by thematic analysis.

## Results

### Section A: Relating to the demographics of the participants

Six hundred and sixty-four online surveys were returned with a response rate of 57%. Upon analysis, it was observed that not all pharmacists worked in pharmacies that provided PHC services, therefore, sections within the online survey were skipped based on applicability. Responses for each question was therefore evaluated individually, resulting in a deviation in the number of responses per for each question.

Participants were predominantly between the ages of 25–44 (54.46%) years old; with 58.61% being females and 41.39% males; practicing across various geographical locations throughout South Africa. Sixty-eight percent of participants were registered with the South African Pharmacy Council for more than 10 years as a pharmacist. All participants were holders of at least a Bachelor of Pharmacy or equivalent degree.

### Section B: Available services delivered and their pricing

Table 1 illustrates the surveyed price [value added tax (VAT) inclusive] that pharmacists charge for services rendered versus the recommended fee a pharmacist may levy stipulated by the South African Pharmacy Council [23]. The USD equivalents are reported based on the exchange rate as at 20 July 2021 [24].

**Table 1:** Fees charged by community pharmacists’ verses the recommended fee by the SAPC for PHC services rendered.

Services being offered by a pharmacist at a community pharmacy	Percentage of community pharmacies that offered the services	Most prevalent fee charged		Recommended fee as per SAPC	
		SA RAND	USD	SA RAND	USD
Blood pressure monitoring	83.28%	ZAR1.00-R50.00	$0.069–3.44	ZAR82.97	$5.71
Blood glucose screening and monitoring	82.07%	ZAR1.00–50.00	$0.069–3.44	ZAR93.59	$6.44
Blood cholesterol and triglyceride screening and monitoring	62.61%	ZAR1.00–50.00	$0.069–3.44	ZAR152.80	$10.51
Immunization	59.57%	ZAR1.00-R50.00	$0.069–3.44	ZAR102.10	$7.02
Reproductive health services (antenatal, family planning, maternity, morning after pill)	57.75%	ZAR51.00-R100.00	$3.51–6.88	ZAR131.06	$9.01
HIV and AIDS testing and pre-test counselling	51,67%	ZAR51.00-R100.00	$3.51–6.88	ZAR663.34	$45.62
Pregnancy screening	50.46%	ZAR1,00-R50,00	$0.069–3.44	ZAR148.73	$10.23
Wound care management	46.81%	ZAR51.00-R100.00	$3.51–6.88	No recommended fee	No recommended fee
Urine analysis	43.77%	ZAR1.00–50.00	$0.069–3.44	ZAR139.69	$9.61
Weight management	41.34%	ZAR1.00-R50.00	$0.069–3.44	No recommended fee	No recommended fee
Drug Utilisation reviews	0%	n/a	n/a	ZAR124.62	$8.57

The general price range that pharmacists levy for a service was found to be between ZAR 1.00–50.00. This was charged based on what each pharmacy felt was an acceptable fee. The most prevalent services offered were blood glucose screening and monitoring, blood pressure monitoring and blood cholesterol and triglyceride screening and monitoring which was administered by a pharmacist 66.15% of the time, registered nurse 55.90% and by an intern or pharmacy assistant under the direct supervision of the pharmacist 23.30% of the time.

Participants were questioned on what would be a suitable and fair reimbursement framework; a consultation fee model, dispensing fee model, and the recommended SAPC fee model were the themes identified. However, 60.61% of participants expressed that a standardized consultation fee model that takes into account what the services entails, resources required and time spent would be the most suitable reimbursement model. Upon thematic analysis as to why participants believed these services should be provided within a community pharmacy, the following themes presented in Table 2 were identified. It was evident that affordability and accessibility were the key drivers for enforcing such a transition in roles.

**Table 2:** Thematic analysis for participants that expressed the benefit of the provision of these services within a community pharmacy (number of responses = 249).

Themes identified	Percentage of participants that expressed the theme
Enhanced accessibility (convenience) and affordability	48.19%
Improved the general health of the community	28.92%
Enhanced the professional profile of pharmacists’ and improves business	14.46%
Improved quality of service	4.02%
Decongestion of High tier of health care and less medical practitioner interaction	4.42%

### Section C: Pharmacists’ perceptions, opinions and their level of readiness

All participants were holders of a Bachelor of Pharmacy degree or equivalent, however, only 8.45% of pharmacists’ had completed a PCDT (Table 3). Despite this, 91.05% were familiar with the standard treatment guidelines which is a list of recommended pharmaceutical and nonpharmaceutical treatments at different levels of care.

**Table 3:** Presents the results from the responses relating to pharmacists’ perceptions, opinions and their level of readiness for implementation of NHI. A: Presents the results for the Yes/No responses. B: Presents the results for the reasons why participant responded "No" to relevant questions.

***A*: *Pharmacist perceptions*, *opinions and level of readiness***			
**Question**		**Responded Yes**	**Responded No**
		**Number of responses for the question (% responses)**	**Number of responses for the question (% responses)**
**1. Have you completed a course in Primary Care Drug Therapy (PCDT) and been issued with a section 22A (15) permit, in terms of the Medicines and Related Substances Act, 101 of 1965?**		53	578
		8.45%	92.19%
**2. Are you familiar with Primary Health Care Standard Treatment Guidelines (STG) and the Essential Medicines List (EML)?**		570	56
		91.05%	8.95%
**3. Do you believe that the NHI rollout is a step in the right direction as South Africa aims to fix gaps in its health care system?**		177	232
		46.21%	53.79%
**4. The roll out of the NHI scheme aims to use pharmacist skills that were previously underutilized to enhance PHC functionality by contributing to the health systems by means of medicine therapy management, new medicines scheme, disease management and monitoring of patients in order to achieve therapeutic success. Do you agree that the proposed change of scope of a pharmacist enables you contribute to improving health care in South Africa with PHC re-engineering with the NHI scheme implementation to enhance service delivery?**		280	135
		74.27%	25.73%
**5. Pharmaceutical literature implies that with adequate training and suitable incentives, a community pharmacist is in a perfect position to provide PHC; however, this requires a criterion shift from focusing on product and sales to becoming more patient centered and focusing on meeting the needs of the community instead. Do you agree with this?**		340	40
		89.24%	10.50%
**6. With the expansion of the scope of practice of a pharmacist does the clinical services offered in a community pharmacy setting fall within the general capabilities of a pharmacist today?**		282	94
		75.00%	25.00%
**7. Do you believe that the implementation of a pharmacist within the NHI infrastructure would enhance pharmacist job performance and satisfaction and stimulate professional morale?**		267	112
		70.45%	29.55%
**8. Based on a scale of 1–5, how confident are you about your profession and capabilities with the intention of expanding the scope of practice of a pharmacist? (1 being very confident, 5 being not confident)**	**Scale**	**Number or responses for the question(n)**	**Percentage response %**
	1	139	36.58%
	2	69	18.16%
	3	88	23.16%
	4	46	12.11%
	5	38	10.00%
***B: Reasons for responding "No" to questions 3 and 4 from Table 3A***			
**Question**	**Responded No**	**Reason for responding No**	**% responses for the question**
	**Number of responses for the question (% responses)**		
**3. Do you believe that the NHI rollout is a step in the right direction as South Africa aims to fix gaps in its health care system?**	232 (53.79%)	*Proposed funding of pooling mechanisms in purchasing health services are too unclear*	*71*.*12%*
		*Lack of information on mechanisms of reimbursement and specifics of involvement of healthcare professionals*.	*68*.*53%*
		*Not enough has been done to change current legislation to cater for the needs of the pharmacist profession as pharmacist scopes of practice are set to change*.	*61*.*64%*
		*It is not the right health care system to implement in South Africa*.	*48*.*71%*
		*Pharmacies lack the equipment*, *facilities and personnel to implement the proposed NHI scheme; and there is very little information available on training and education of personnel to perform these task*.	*46*.*12%*
**4. The roll out of the NHI scheme aims to use pharmacist skills that were previously underutilized to enhance PHC functionality by contributing to the health systems by means of medicine therapy management, new medicines scheme, disease management and monitoring of patients in order to achieve therapeutic success. Do you agree that the proposed change of scope of a pharmacist enables you contribute to improving health care in South Africa with PHC re-engineering with the NHI scheme implementation to enhance service delivery?**	135 (25.73%)	*Not all pharmacists have the experience to perform these additional services and therefore additional training is required (e*.*g*., *PCDT is required)*	*60*.*74%*
		*Monetary input from individuals that remain on privately funded medical schemes may not be sufficient to support the retail pharmacy*	*55*.*56%*
		*It is better for retail pharmacies to be contracted as an additional medicine distribution point under the NHI to provide these services and more economical so that these pharmacies can still sustain themselves financially*	*54*.*81%*
		*It is just not a sustainable option in moving PHC in South Africa into a positive direction*.	*37*.*78%*
		*It would be difficult to ensure service delivery is equitable*	*37*.*04%*

It was revealed that 53.79% of pharmacists believed that the NHI will not navigate SA in the right direction with the primary reason being lack of information on mechanisms of reimbursements (68.53%) and the proposed pooling mechanisms for funding being too elusive (71.12%). Upon thematic analysis of opened ended responses (n = 78) as to why NHI will not be the right model, it was found that the lack of reimbursement and sustainability strategies (38.55%); lack of information on the public-private transition (38.55%) and mismanagement of funds by government (22.89%) were the 3 emerging themes.

Majority of pharmacists (74.27%) believe that the expansion of a pharmacists role within the NHI framework will be beneficial in improving the functionality and efficiency of the PHC environment; with 89.24% believing that further training is required in order to shift pharmacists roles from product-centred to patient-centred. Table 3, also indicates that 75% of pharmacists believe that the requirements for a pharmacist to render PHC services fall within the scope of practice of a pharmacist, but only 36.58% are completely confident in their own professional capabilities. A large percentage (70.45%) of pharmacists advocate that the transition to patient-centred roles will positively enhance the morale of the pharmacy profession.

## Discussion

In this study we sought to determine current pricing and reimbursement models used for the provision of PHC within a community pharmacy; and to evaluate suitable reimbursement for the provision of such services by a community pharmacist. We also assessed the readiness and perceptions of pharmacists to take on the envisioned expanded roles and responsibilities in an integrated health care system such as NHI. The discussion below highlights the key findings of this study.

It was observed that not all pharmacies offered PHC services. However, it may be advantageous to standardise the provision of such services within a community pharmacy environment. Amongst them being the advantage of being able to act immediately to improve patient outcomes, particularly patients on chronic medication based on tests performed by the pharmacist; this exhibited the need for such services to be provided. Furthermore, the provision of these services in a community pharmacy setting allowed patients to have better access and implement necessary health interventions much sooner. The top 3 most prevalent services i.e. blood pressure, blood glucose and blood cholesterol that were offered within pharmacies are essential services that directly affect the prevalence of non-communicable diseases in SA. Therefore, the increase in access to such services may be beneficial in ensuring appropriate chronic care detection and management and may positively contribute in reducing the quadruple burden of disease that SA faces. It was observed in this study that pharmacists were the primary providers of these services in community pharmacies; further substantiating that pharmacists’ already have the skillset to offer these services. The advantages of the above findings were similarly identified in studies conducted in both Canada and the United states [17, 25].

There is also the potential barrier that there are little financial incentives for a pharmacist to provide so many clinical services when compared to other medical professionals. This was also observed in a study conducted in Iran [26]. Despite this concern of inadequate financial incentives, the study found that majority of pharmacists were levying their services at a price lower than the recommended SAPC fee structure. It is therefore important for policy makers and reimbursors to standardise reimbursement structures with taking into consideration the resources required to offer these services and running operational cost of a pharmacy to ensure that pricing is profitable and fair. These policies will in turn enhance confidence in pharmacists as one of the main concerns that participants echoed is the need for adequate reimbursement strategies to confidently transition into these new roles.

It was identified that other services such as DURs, PrEP, oncology mixing were not currently offered by any of the pharmacies. The provision of such services by a community pharmacist may be beneficial in the advising of newly diagnosed conditions and medicines, clarifying treatments prescribed and administration, decreasing the symptoms and long term complications associated with chronic conditions, identify medication taking problems, provision of additional information and instructions for better self-management and to promote better lifestyle changes [27, 28]. The inclusion of oncology mixing within a community pharmacy environment also enables patients and caregivers to access their medicine at a more conveniently and the necessary supportive therapy guidance [29]. Furthermore, due to the high prevalence of HIV and AIDS in South Africa [30], increased access to prevention regimens such as PrEP would be extremely beneficial. Therefore, there is room for the expansion of services that can be provided by a community pharmacist.

Upon thematic analysis the key reason participants advocated for the provision of the services mentioned in the study within a community pharmacy was predominantly due to the increase in accessibility and affordability for patients. This offers patients the convenience of visiting their community pharmacy and the benefit of regular health status check-ups at a more affordable cost. The second most prevalent theme identified was that the provision of such services will improve the overall health of the community. These two themes work synergistically, as the community has improved access to care and health information; subsequently ensuring that they can make the necessary preventative health decision, resulting in better patient outcomes. This finding was similarly observed in studies conducted in Indonesia, Pakistan and the United Kingdom [31–33].

A larger percentage of participants were knowledgeable on the use of the South African standard treatment guidelines. This may provide evidence that pharmacists’ are adequately educated to provide PHC services within their scope; as well as are sufficiently trained in ensuring safe and rational drug use within their practice. Currently, in SA a pharmacist needs to complete an additional PCDT courses in order to diagnose and prescribe medicine according to and approved list. The completion of a PCDT course allows a pharmacist to provide an advanced service package (minor ailments, immunisation, family planning and comprehensive wellness screening). Therefore, such expertise may be beneficial in transitioning into these new roles as pharmacists can initiate immediate health interventions. However, only a very small percentage of participants completed a PCDT course. This may be a potential barrier in determining the accreditation required for the provision of these services. Policy makers have to take into account the requirements and educational background required for a pharmacist to render services at a PHC level; and is it sufficient to only have a Bachelor of Pharmacy or equivalent degree or should courses like PCDT be completed. Tertiary institutions should be encouraged to offer courses such as PCDT within their curriculum; so that pharmacists can specialize in individualised avenues of care and are adequately exposed to clinical practices. Statutory councils such as the SAPC should fast-track recognition of these specialised fields of practices for pharmacists for quicker accreditation. Currently in SA, the SAPC has introduced a mandatory continuous professional development (CPD) program; this can also be used as a platform to enhance the needed professional development, technical capacity or upskilling of a pharmacist professional capabilities.

It was also identified that even though a majority of participants (75%) believed that the requirements for a pharmacist to render PHC services fall within the scope of practice of a pharmacist, only 36.58% are completely confident in their own professional capabilities. This gap may also reinforce the necessity for further training, as pharmacists may be equipped to perform these services but additional training will enhance professional confidence for a smoother transition into these new roles. There is also a need to enhance pharmacists’ roles in the health care team with other healthcare providers, the public and government authorities. Therefore, appropriate referral pathways should now consider community pharmacists as the first call prior to upscaling to higher levels of care.

Traditionally pharmacists’ take on a primary role of ’custodians of medicine’ [34]. It has been found that in lower-income countries, majority of the population utilizes public healthcare facilities, resulting in them being overburdened and understaffed, with a heavy workload of medicine dispensing, distribution and administration taken up by pharmacists and pharmacy personnel [35]. Therefore, with standardised guidelines and reimbursement policies community pharmacist can be introduced within the PHC service package, alleviating the strain on the health care system.

The findings of this study also exhibits pharmacists willingness to take on these new professional roles as 70,45% believe that their incorporation within the NHI infrastructure would enhance job performance and satisfaction. Therefore, repositioning pharmacist to holistically apply their skills and expand their professional capabilities not only stimulates job satisfaction but results in better work engagement; directly affecting performance which affects patient care outcomes [36].

Another important observation was that a majority of participants did not believe that NHI was the right model to adopt in SA. The key reason being a lack of information on policies. It was also evident that a large concern was the lack of information particularly on reimbursement strategies; this highlights the importance of this study further. Another concern was the transition between public-private partnerships; more information is required by role players on how the government plans to upscale the current state facilities in order to ensure that an equitable healthcare system is implemented. Both concerns are related to the lack of knowledge and information on policy implementation. This is an important concern that requires extensive consideration as role players influence the successful implementation of the policy put into practice. Understanding the dimensions of the policy and its implementation can aid those responsible to implement it more effectively [37]. Therefore, it is essential that policy makers illustrate the various roles of different role players and their mechanisms of reimbursement to promote better professional buy-in. Another concern was the mismanagement of funds by the government. "Good governance" enables appropriate allocation of resources to ensure efficient policy roll-out. With that said there is a rising trend in the mis-management of funds in LMICs such as South Africa. In a study that assessed the influence of corruption on health care service delivery in LMICs of south and south-east Asia, it was observed that the prevalence of corruption resulted in poor health care provider reimbursement and inadequate supply of resources. This led to high out-of-pocket expenditure, lack of trust in the health care system and poor quality of services [38]. Therefore, it is understandable that private sector pharmacist may be reluctant in joining the public- private partnership. However, in order to alleviate these concerns policy makers should be transparent on funding mechanism and the implementation of strategic pooling to avoid mismanagement of funds.

Overall the study found that with adequate training and understanding of the full requirements of services to be rendered, pharmacist are willing to transition into these new roles to ensure that SA has a PHC system that runs more efficiently.

### Limitations

This study would have benefitted from using pricing data for each service. However, due to the fact that not all pharmacies offered a full complement of PHC services, it makes it difficult to conclusively compare prices. It was also found that the study would of benefitted from correlating geographical diversity, socio-demographics and experiential data of participants. Future studies would benefit from investigating the link between the findings in section A results to the perceptions, opinions and level of readiness of pharmacists. Furthermore, an additional limitation is that the findings of this study reports descriptive data; future studies would benefit from more inferential statistics to further evaluate the findings. With that said the findings does provide foundational evidence for future consideration in constructing reimbursement frameworks.

## Conclusion

Universal health coverage aims to reduce the gap between availability and accessibility of health services in public and private sectors in LMICs such as South Africa. This study highlights the perceptions and level of readiness of pharmacists, so that they could more efficiently contribute to PHC re-engineering to attain this goal. The results of this study emphasized that the inclusion of a pharmacist in the PHC service package is beneficial in reducing strain on the health care system; but also raised concerns regarding the standardization of guidelines (practice and reimbursement) for the provision of such services under NHI. This study also indicated that current practice for a role transition by pharmacists may result in a disparity in professional capabilities and requirements. In order to correct this disparity, policy and practice changes need to be made collaboratively between policy makers and role players to promote better synergism within the healthcare system. Therefore, future research and policy should be focused on defining a comprehensive package of services that can be provided by a pharmacist within a PHC environment. This study also highlights the fundamental value of introducing a pharmacist as a PHC provider as well as potential barriers that may be experienced when attempting to adopt these new roles. Therefore, future research should focus on the standardization of clinical guidelines and protocols. Furthermore, policymakers must take into consideration fair reimbursements for the rendering of these services to be sustainable.

## Supporting information

S1 Table Roles and reimbursement of pharmacist as South Africa transitions towards Universal Health Coverage (UHC): Survey. (DOCX)

S1 Data (XLSX)

## Abbreviations

NHI: National Health Insurance
PHC: Primary Health Care
UHC: Universal Health Coverage
