# Supplementary material for: Roles and reimbursement of pharmacists as South Africa transitions towards Universal Health Coverage (UHC): An online survey-based study
Source: PLoS One. 2021 Sep 23;16(9):e0257348. doi: 10.1371/journal.pone.0257348 (PMC8459985; doi:10.1371/journal.pone.0257348)
Supplement: S1 Table — (DOCX) [file pone.0257348.s001.docx]

# **S1 Table. Roles and reimbursement of pharmacist as South Africa transitions towards Universal Health Coverage (UHC): Survey**

## **Invitation for Participation in Study**

Dear Colleague (Healthcare team member),
You are being invited to consider participating in a study that we are conducting at your facility by Ms VC Naidoo, Dr V Bangalee  and Prof F Suleman from the School of Health Sciences at the University of KwaZulu- Natal, South Africa.

OVERVIEW OF THE STUDY

The South African government has begun developing  new healthcare policies and regulations for moving towards Universal Health Coverage (UHC) through the implementation of National Health Insurance (NHI). UHC aims to improve health in low and middle-income countries, like South Africa. The conventional role of a pharmacist practicing in South Africa is set to change as the public-private partnership aims at employing other skills that have been underutilized, thus extending the scope of practice of a pharmacist. Currently, pharmacists are remunerated via a dispensing fee based on the Single Exit Price (SEP). However, with the NHI roll-out remuneration for pharmacists should take into account the services that are, and can be provided by a pharmacist to enhance the functionality of the PHC system. This in turn aims to improve the accessibility, affordability and availability of medicines and health services to all South African citizens. This survey is a baseline study that will form the directive for further research; as it aims to determine which PHC services are already available at retail pharmacies and what are the costs of these services. This will provide evidence for future reimbursement policies in the NHI implementation. The survey also aims to gain an understanding of pharmacist perceptions on the NHI roll-out that is set to change their scope of practice.

STUDY PROCEDURES

You are invited  to participate in a survey which will take  approximately 20 minutes to complete.

RISKS

There are no known risks to you or your organisation associated with participating in the study.

BENEFITS

There are no direct benefits for participants. However, this study may provide you with an opportunity to contribute towards improving reimbursement policies of healthcare professionals in South Africa.

VOLUNTARY PARTICIPATION

Participation in the study is completely voluntary. You may refuse to participate or may withdraw from the study at any time.

CONFIDENTIALITY

The information collected in the study will be used for research purposes only. The study will be completely confidential, and your name will not appear anywhere in the study. A pseudonym will be used instead of your name and efforts will be made not to disclose your identity. Your participation and input will be strictly confidential.

This study has been ethically reviewed and approved by the UKZN Biomedical research Ethics Committee (BREC Ref No: BE625/17).

In the event of any problems or concerns/questions you may contact the researchers on 031 260 7741 or Bangalee@ukzn.ac.za / vcampbelln@gmail.com or the UKZN Biomedical Research Ethics Committee, contact details as follows:

BIOMEDICAL RESEARCH ETHICS ADMINISTRATION
Research Office, Westville Campus
Govan Mbeki Building
Private Bag X 54001
Durban
4000
KwaZulu-Natal, South Africa
Tel: 27 31 2604769 - Fax: 27 31 2604609
Email: BREC@ukzn.ac.za

Your participation would be greatly appreciated! Thank you for your consideration and time.

| **Informed Consent (Tick all relevant boxes)** |
| --- |
| Answer Choices |
| I confirm that I have been informed about the study by Ms V. Naidoo, Dr. V Bangalee and Prof F Suleman: The Role of  the Pharmacist as Primary Health Care is being  re-engineered during the Implementation of the National Health Insurance. |
| I understand the purpose and procedures of the study. |
| I have been given an opportunity to answer questions about the study and have had answers to my satisfaction. |
| I declare that my participation in this study is entirely voluntary and that I may withdraw at any time without affecting any of the benefits that I usually am entitled to. |
| If I have any further questions/concerns or queries related to the study I understand that I may contact the researcher. |
| If I have any questions or concerns about my rights as a study participant, or if I am concerned about an aspect of the study or the researchers then I may contact BREC or Dr Varsha Bangalee or Ms Vivian Naidoo. |

| **Agreement to Participate in the Study** |
| --- |
| Answer Choices |
| I agree to participate. |
| I do not agree to participate. |

Section1: Demographic data

Question 1

| **What is your age?** |
| --- |
| Answer Choices |
| 18-24 |
| 25-34 |
| 35-44 |
| 45-54 |
| 55-64 |
| 65+ |

Question 2

| **What is your gender?** |
| --- |
| Answer Choices |
| Female |
| Male |

Question 3

| **What is you geographic area of practice? (Province and district)** |
| --- |

Question 4

| **Years of practice as a registered pharmacist?** |
| --- |
| Answer Choices |
| 0-2 years |
| 3-5 years |
| 6-10 years |
| more than 10 years |

Question 5

| **Languages spoken** |
| --- |

Question 6

| **Auxiliary courses** | **degrees or diplomas completed that are pharmacy related (Name of course and year completed)** |
| --- | --- |

Section 2: Service delivery

Question 7

| **Have you completed a course in Primary Care Drug Therapy (PCDT) and been issued with a section 22A (15) permit, in terms of the Medicines and Related Substances Act, 101 of 1965 ? (Please state year awarded)** |
| --- |
| Answer Choices |
| Yes |
| No |
| Please specify year awarded. |

Question 8

| **Are you familiar with Primary Health Care Standard Treatment Guidelines (STG) and the Essential Medicines List (EML) ?** |
| --- |
| Answer Choices |
| Yes |
| No |

Question 9

| **Please tick the services being offered at the pharmacy you are currently practicing at.** |
| --- |
| Answer Choices |
| Blood glucose screening and monitoring |
| Blood cholesterol and triglyceride screening and monitoring |
| Urine analysis |
| Blood pressure monitoring |
| HIV and AIDS pre-test counselling, testing and post-test counselling |
| Pregnancy screening |
| Oncology mixing |
| Reproductive health services (antenatal, family planning, maternity, morning after pill) |
| Immunization |
| Weight management |
| Wound care management |

Question 10

| **What is the fee charged for each of the services offered in your pharmacy? (Please specify in ZAR)** |
| --- |
| Answer Choices |
| Blood glucose screening and monitoring |
| Blood cholesterol and triglyceride screening and monitoring |
| Urine analysis |
| Blood pressure monitoring |
| HIV and AIDS pre-test counselling, testing and post-test counselling |
| Pregnancy screening |
| Oncology mixing |
| Reproductive health services (antenatal, family planning, maternity, morning after pill) |
| Immunization |
| Weight management |
| Wound care management |

Question 11

| **What do you think are the key benefits from these services being offered at your pharmacy?** |
| --- |

Question 12

| **Who is conducting these services in your pharmacy?** |
| --- |
| Answer Choices |
| Pharmacist |
| Registered nurse |
| Intern under supervision of pharmacist |
| Pharmacy assistant under the supervision of pharmacist |

Question 13

| **How were the fees for these services calculated?** |
| --- |

Section 3: Pharmacist perceptions and service reimbursement

Question 14

| **Do you believe that the NHI rollout is a step in the right direction as South Africa aims to fix gaps in its health care system?** |
| --- |
| Answer Choices |
| Yes |
| No |

Question 15

| **If you have answered 'NO' please tick the relevant options for your response.** |
| --- |
| Answer Choices |
| Lack of information on mechanisms of reimbursement and specifics of involvement of healthcare professionals. |
| It is not the right health care system to implement in South Africa. |
| Not enough has been done to change current legislation to cater for the needs of the pharmacist profession as pharmacist scopes of practice are set to change. |
| Pharmacies lack the equipment, facilities and personnel to implement the proposed NHI scheme; and there is very little information available on training and education of personnel to perform these task. |
| Proposed funding of  pooling mechanisms in purchasing health services are too unclear |
| Other (please specify) |

Question 16

| **The roll out of the NHI scheme aims to use pharmacist skills that were previously underutilized to enhance PHC functionality by contributing to the health systems by means of medicine therapy management, new medicines scheme, disease management and monitoring of patients in order to achieve therapeutic success. Do you agree that the proposed change of scope of a pharmacist enables you contribute to improving health care in South Africa with PHC re-engineering with the NHI scheme implementation to enhance service delivery?** |
| --- |
| Answer Choices |
| Yes |
| No |

Question 17

| **Pharmaceutical literature implies that with adequate training and suitable incentives, a community service pharmacist is in a perfect position to provide PHC; however, this requires a criterion shift from focusing on product and sales to becoming more patient centred and focusing on meeting the needs of the community instead. Do you agree with this? *** |
| --- |
| Answer Choices |
| Yes |
| No |
| If you answered NO, please explain. |

Question 18

| **What in your opinion would be an appropriate reimbursement fee model for pharmacist skills used to perform PHC  services? (i.e. in comparison to the old health care system that utilities the dispensing fee)** |
| --- |

Question 19

| **With the expansion of the scope of practice of a pharmacist does the clinical services offered in a pharmacy setting fall within the general capabilities of a pharmacist today?** |
| --- |
| Answer Choices |
| Yes |
| No |

Question 20

| **From the list below, how confident are you about your profession and capabilities with the intention of expanding the scope of practice of a pharmacist. (1 being very confident, 5 being not confident)** |
| --- |
| Answer Choices |
| 1 |
| 2 |
| 3 |
| 4 |
| 5 |

Question21

| **Do you believe that the implementation and using of the NHI infrastructure would enhance pharmacist job performance and satisfaction and stimulate professional morale?** |
| --- |
| Answer Choices |
| Yes |
| No |
